# The Hyperphagia Questionnaire: Insights From a Multicentric Validation Study in Individuals With Prader Willi Syndrome

**DOI:** 10.3389/fped.2022.829486

**Published:** 2022-02-14

**Authors:** Maria Rosaria Licenziati, Dario Bacchini, Antonino Crinò, Graziano Grugni, Danilo Fintini, Sara Osimani, Letizia Ragusa, Michele Sacco, Lorenzo Iughetti, Luisa De Sanctis, Adriana Franzese, Malgorzata Gabriela Wasniewska, Maria Felicia Faienza, Maurizio Delvecchio, Concetta Esposito, Giuliana Valerio

**Affiliations:** ^1^Department of Neurosciences, Obesity and Endocrine Disease Unit, Santobono-Pausilipon Childrens Hospital, Naples, Italy; ^2^Department of Humanistic Studies, University of Naples “Federico II”, Naples, Italy; ^3^Reference Center for Prader-Willi Syndrome, Research Institute, Bambino Gesù Hospital, Rome, Italy; ^4^Department of Auxology, Istituto di Ricerca e Cura a Carattere Scientifico Istituto Auxologico Italiano, Verbania, Italy; ^5^Department of Pediatrics, Scientific Institute San Raffaele, Milan, Italy; ^6^Oasi Research Institute-IRCCS, Troina, Italy; ^7^Pediatric Unit Scientific Institute “Casa Sollievo Della Sofferenza” San Giovanni Rotondo (FG), San Giovanni Rotondo, Italy; ^8^Pediatric Unit, Department of Medical and Surgical Sciences for Mothers, Children and Adults, University of Modena and Reggio Emilia, Modena, Italy; ^9^Department of Public Health and Pediatric Sciences, University of Torino, Turin, Italy; ^10^Department of Traslational Sciences, University of Naples Federico II, Naples, Italy; ^11^Department of Human Pathology of Adulthood and Childhood, University of Messina, Messina, Italy; ^12^Department of Biomedical Sciences and Human Oncology, Pediatric Unit, University of Bari “Aldo Moro”, Bari, Italy; ^13^Metabolic Disorders and Genetics Unit, “Giovanni XXIII” Children's Hospital, Azienda Ospedaliera Universitaria (A.O.U.) Policlinico di Bari, Bari, Italy; ^14^Department of Movement Sciences and Wellbeing, Parthenope University of Naples, Naples, Italy

**Keywords:** hyperphagia, Prader-Willi syndrome, multicentric study, weight status, Genetic Obesity, assessment

## Abstract

**Background/Objectives:**

The present study aimed to validate the Italian version of the Hyperphagia Questionnaire (HQ), a 11-items questionnaire developed to assess hyperphagia in individuals with Prader-Willi syndrome (PWS). This is a complex neurodevelopmental disorder characterized by endocrine dysfunction, hypotonia, intellectual disability, psychiatric disorders and obesity.

**Methods:**

Parents of 219 individuals with PWS (age range 3–54 years; M_age_ = 17.90; 108 Males), recruited in 12 hospitals in Italy responded to HQ during routine visits. In function of the level of analyses the sample was divided into two subgroups (<18> years) or into four age-subgroups (2.5–4.5; 4.5–8; 8–18; >18 years) corresponding to different clinical stages.

**Results:**

Confirmatory factor analysis (CFA) confirmed the three hyperphagic subdimensions of the original structure (behavior, drive, and severity), but one item was dropped out, reducing the final version to 10 items. Using multi-group CFA, HQ showed satisfactory indexes of measurement invariance by age. Good indexes of internal consistency (Cronbach's alpha and McDonald's Omega coefficients) were found for each subdimension. The three hyperphagia subdimensions positively converged with other food-related measures: emotional overeating, food enjoyment, food responsiveness, and satiety responsiveness. A significant increase of all hyperphagic subdimensions was found across age groups. Higher hyperphagic levels were found in participants with higher body mass index. Hyperphagic drive differently increased in function of the interaction between age and underlying genetic mechanisms.

**Conclusion:**

The Italian version of the HQ is a psychometrically valid and reliable instrument for assessing hyperphagia in individuals with PWS. This tool may prove useful to evaluate the efficacy of pharmacologic and rehabilitative treatments.

## Introduction

Prader–Willi syndrome (PWS) is a rare genetic disorder with a birth incidence ranging from 1/10,000 to 1/30,000 (1) equally distributed by sex and ethnicity (2). PWS is caused by the *de novo* absence of paternally-expressed, imprinted genes on chromosome 15q11–13. This loss of activity may be determined by three major genetic mechanisms: (i) microdeletion (DEL) of the paternally inherited chromosome (60–70% of cases); (ii) maternal uniparental disomy (mUPD) subsequent to trisomic rescue (30–40% of cases); (iii) imprinting center defect (IC) or translocation (3–5% of cases) (3).

PWS is a multifaceted neurodevelopmental disorder characterized by endocrine dysfunction, hypotonia, intellectual disability, psychiatric disorders and obesity (1, 4). Psychiatric disorders include a variety of symptoms such as anxiety and depression (5), affective disorders (6), temper tantrums (7), obsessive-compulsive behaviors (8), self-injurious behaviors (9), autistic behaviors (10), and hyperactivity (11).

A hallmark of PWS is represented by hyperphagia, commonly defined by an excessive hunger and abnormal intake of food. Forms and severity of hyperphagia in individuals with PWS vary across the life-span. According to Miller et al. (12) seven developmental phases characterize the clinical course of PWS; specifically, four sub-phases concern specific nutritional phenotypes. In a first period (phase 2a) from 2.1 to 4.5 years children begin to develop weight gain in absence of additional food consumption. In a second period (phase 2b) from 4.5 to 8 years children continue to increase weight and begin to develop hyperphagia with some satiety. In a third period (phase 3), from 8 years through adolescence and adulthood, hyperphagia continue to worsen, reaching the peak. In the fourth period (phase 4), occurring in adulthood, appetite is impossible to satisfy for some individuals with PWS, while it becomes satiable for others, since the hyperphagic drive lessens. However, the age at the onset of each phase should not be strictly considered, because the age of transition from one nutritional phase to the succeeding one can vary among individuals as well as the severity of hyperphagia.

To investigate hyperphagia in individuals with PWS posits challenging issues for many reasons. A first critical question concerns the operationalization of the construct of hyperphagia. As Yanovsky (13) pointed out, a shared definition is far to be reached and consequently there is no agreement on the measurement methods. Moreover, evidence supporting the role of specific genes on neural circuits that control features of food intake and food reward, as well as the effective treatments to contrast hyperphagia, are inconclusive (13). It should also be considered that, beyond genetic diseases, hyperphagic behavior can be associated to obesity, binge eating disorder, and emotional overeating (14).

The most used tool for assessing hyperphagia in PWS is the Hyperphagia Questionnaire (HQ) specifically designed by Dykens et al. (15) to measure symptoms of food related preoccupations and behaviors. The HQ is addressed to caregivers of individuals with PWS. Original version is composed by 11 items aimed to investigate the perseveration about seeking food (bargaining, asking/talking about food), the intensity of emotions around food (getting upset about being denied a desired food), and the severity of thoughts and activities around food in interfering with normal activities. Based on 11 item version, the authors distinguished, through an explorative factorial analysis, three main factors labeled as hyperphagic behavior, hyperphagic drive, and hyperphagic severity. The measure has been used in several observational studies in individuals with PWS (8, 10, 16, 17) and in clinical trials (18–21) in order to detect the efficacy of treatment. The questionnaire was also used with adaptations by researchers. Some authors (5, 22), for instance, used both a total score and three subscores, others focused on the three subscores (15, 16, 19). Moreover, qualitative and quantitative evidences support the use of the total score obtained from a 9-item version for the assessment of food-seeking behaviors in PWS clinical trials (HQ-CT) (21).

Whether sex and genetic subtypes differentially affected the seriousness and the onset of hyperphagia remain challenging questions (23). The little research examining gender differences in the severity of eating maladaptive behaviors in individuals with PWS evidenced inconclusive results. In general, females were found to show more behavioral problems than males (24) whereas Gito et al. (25) found an interesting interaction between gender and genetic subtypes: in subgroup with deletion, males exhibited more food related problems than females, whereas in mUPD subgroup females presented higher food related problems than males. However, findings concerning the association between genetic subtypes in PWS and food-related problems are inconclusive. For instance, higher food-related problems were found in individuals with microdeletion than those with mUPD (19) while Key et al. (26) found a longer fixation on food stimuli using an eye tracking experimental procedure (a proxy measure of problematic food interest), in mUPD subtype than the deletion subgroup.

Also the association between obesity and PWS requires further investigation. It is undeniable that obesity is a common characteristic of PWS caused by the combined excessive eating and reduced physical activity even though, in her seminal study, Dykens (15) found an association between weight status and hyperphagia in children but not in adults. Moreover, in the last years, effective drug treatments, behavioral-based rehabilitative programs, and a strict environmental control evidenced a high likelihood to reduce the obesity-related risk (27, 28).

Therefore, the aims of the current research were:

(i) To validate the hyperphagia questionnaire (15) in individuals with PWS within the Italian context, testing construct and convergent validity, and measure invariance. To our knowledge, this is the first systematic investigation carried out in the Italian context. We expected a confirmation of the three factors structure of the HQ as found by Dykens et al. (15) a positive convergence with proximal food-related dimensions such as food responsiveness, emotional overeating, food enjoyment, and a negative convergence with satiety responsiveness.
(ii) To compare the hyperphagic scores in individuals with PWS by gender, age, obesity and genetic subtypes. We expected differences in hyperphagic scores by age groups with a peak, according to the theory, between 8 and 18 years. We did not advance specific hypotheses regarding differences by sex, weight status and genetic subtypes given to the limited literature at this regard.

## Method

### Participants

Parents or primary guardians of persons with confirmed genetic diagnosis of PWS (age range 3–54 years) were consecutively invited to participate to this multicentric and cross-sectional study. They were recruited in 12 hospitals distributed across northern (Milan, Turin, Verbania), central (Rome and Modena), and Southern Italy (Bari: two sites, San Giovanni Rotondo, Naples: two sites, Messina, and Troina).

All participants were invited to take part to the study during routine visits between January and December 2019. The study was approved by the Ethical Committee of the Santobono-Pausilipon Hospital (leader center of the study) and by the Review Board of the Genetic Obesity Study Group of the Italian Society of Pediatric Endocrinology and Diabetology. Written informed consent was obtained from all participants in accordance with the revised version of the Helsinki Declaration regarding research involving human subjects.

Fifteen parents refused to participate to the study. Finally, the collected data regarded 219 individuals with PWS (108 M and 111 F; age range 2.5–54). In function of the level of analyses, the sample was considered as a whole, or it was divided into subgroups. Two age subgroups (children <18 years vs. adults >18 years) were used to validate HQ. Four age subgroups (group A: 2.5–4.5 years; group B: 4.5–8 years; group C: 8–18 years; group D: >18 years) were created to investigate how food behavioral problems in individuals with PWS differ across the four main developmental nutritional periods (12, 29).

### Measures

#### Anthropometry and Genetic Characteristics

Anthropometric and sociodemographic measures were collected during the visit. Body weight was determined to the nearest 0.1 kg on properly calibrated standard beam scales, in minimal underclothes and no shoes. Height was measured to the nearest 0.5 cm on standardized, wall-mounted height boards according to standardized procedures. The BMI was calculated as weight divided by square of height (kg/m^2^). Absolute values of BMI were employed in adults (>18 years) to define normal-weight (NW: BMI 18–24.9), Overweight (OW: BMI 25–29.9), Obese (OB: 30–34.9), Severe Obese (SOB: >35) (30). The BMI standard deviation score (BMI-SDS) was used in individuals <18 years according to the WHO reference curves (31, 32). NW was defined as BMI-SDS between−1 and +1, OW as BMI-SDS between 1.01 and 2, OB as BMI-SDS between 2.01 and 3, and SOB as BMI-SDS >3.

All individuals received genetic diagnosis of PWS even though 8 individuals had a positive methylation test, but the underlying genetic defect was not identified. Sample's characteristics related to age, sex, genetic and weight status are reported in the Table 1.

**Table 1:** Characteristics of 219 individuals with PWS participating in the study across age groups.

	**Age groups**				
**Characteristics**	**Group A**	**Group B**	**Group C**	**Group D**	**Total (N. 219)**
	**2.5–4.5 years (N. 20)**	**4.5–8 years (N. 32)**	**8–18 years (N. 82)**	**>18 years (N. 85)**	
*Age* (Mean ± SD)	3.34 ± 0.73	6.5 ± 1.10	12.65 ± 2.81	30.73 ± 10.08	17.90 ± 12.53
**Gender**					
Female	10 (50%)	16 (50%)	37 (45.1%)	48 (56.5%)	111 (50.7%)
Male	10 (50%)	16 (50%)	45 (54.9%)	37 (43.5%)	108 (49.3%)
**Body weight status^a^**					
Normal weight (NW)	13 (65%)	13 (40.6%)	21 (25.6%)	9 (10.6%)	56 (25.6%)
Overweight (OW)	3 (15%)	6 (18.8%)	20 (24.4%)	15 (17.6%)	44 (20.1%)
Obese (OB)	4 (20%)	6 (18.8%)	23 (28%)	36 (42.4%)	69 (31.5%)
Severe Obese (SOB)	0 (0%)	7 (21.9%)	18 (22.0%)	25 (29.4%)	50 (22.8%)
**PWS genetic subtypes^b^**					
Deletion (DEL)	6 (31.6%)	11 (35.5%)	37 (48.7%)	57 (67.1%)	111 (52.6%)
Maternal UPD (mUPD)	12 (63.2%)	16 (51.6%)	31 (40.8%)	28 (32.9%)	87 (41.2%)
Imprinting (IC)	1 (5.2%)	4 (12.9%)	8 (10.5%)	0	13 (6.2%)
MD^c^	1	1	6		8

a *Body weight status was evaluated from BMI- SDS for individuals <18 and from BMI for individuals >18*.

b *Percentages were computed excluding missing data (MD)*.

c *Missing Data*.

#### Hyperphagia Questionnaire

We used the 11-item version of the Hyperphagia Questionnaire (15). Items refer parental reports to food-related preoccupations, severity of these concerns, and atypical food-related behaviors. The response format was on a 5-point scale (scored 1–5). The timeframe for parents' recall of the hyperphagia symptoms varied across the HQ questions, according to the original version. Questionnaire was filled in a written form by parents. An assistant researcher was present during administration to provide assistance if required. A procedure of translation and backtranslation was used to ensure the linguistic equivalence of each item into the Italian language. The steps were: (i) a native Italian speaker fluent in English translated from English into Italian; (ii) a native English speaker fluent in Italian translated into English; (iii) controversial issues were discussed within the research group with the counseling of a second native English fluent in Italian. The Italian translation is reported in Supplementary Table 1. The original factor analysis identified three independent factors: Hyperphagic Drive (5 items, e.g., *how persistent in asking for food*); Hyperphagic Behaviors (4 items, e.g., *stealing food*), and Hyperphagic Severity (2 items, e.g., *extent that food interferes with everyday functioning*).

#### Convergent Measures

In order to establish convergent validity of the questionnaire we invited care providers to respond to a set of 10 items drawn from Children's Eating Behavior Questionnaire (33). Despite the questionnaire was developed for children, items were evaluated fit also for adults (34). Four sub-dimensions were used: emotional eating (2 items: e.g., *My child eats more when anxious*), food responsiveness (3 items, e.g., *If allowed to, my child would eat too much*), satiety responsiveness (2 items: e.g., *My child gets full up easily*- reversed), food enjoyment (3 items: e.g., *My child loves food*). Items were rated by care providers on a 5-point scale (1 = never to 5 = often).

### Statistical Analyses

Before testing the factorial validity of the HQ, we conducted inter-item correlations to examine the extent to which scores on one item were related to scores on all the other items in a scale. A correlation value >0.30 was used as the cut-off value above which items on a scale can be considered as assessing the same content (35). Then, we ran a Confirmatory Factor Analysis (CFA) to examine the original theoretical latent factor structure of the measure against alternative models. More specifically, fit statistics for the three-factor model were compared to those of a one-factor model. The chi-square statistic divided by the degrees of freedom (χ^2^/df ≤ 3), the comparative fit index (CFI), the root mean square error of approximation with associated 90% confidence intervals (RMSEA), and the standardized root mean square residual (SRMR) were used to evaluate the adequacy of models to data (36). Acceptable model fit was defined following the criteria provided by Hu and Bentler (37): CFI ≥ 0.95, RMSEA ≤ 0.06, 90% CI ≤ 0.06, and SRMR ≤ 0.08. The chi-square difference test for nested models was used to compare the three-factor model with the one-factor model. Significant results for the χ^2^ difference test indicate that the model with smaller χ^2^ has a statistically better fit. The internal consistency of the resulting HQ factors was computed using Cronbach's alpha and McDonald's omega.

As a second step, we investigated the invariance of the factor structure across age groups (children ≤ 18 years old; adults >18 years old) using a multiple-group structural equation model. A series of chi-square difference tests comparing fit statistics of hierarchical models were performed in order to investigate: configural invariance, with factor loadings, intercepts, and error variances free to vary across subsamples; metric invariance, with invariant factor loadings across the two subsamples, and intercepts and error variances allowed to vary; scalar invariance, with invariant factor loading and intercepts across subsamples, and error variances free to vary.

Third, once the HQ factor structure was identified, we tested the hypothesized convergence. Using the mean scores reported by each individual in HQ factors, we investigated, running zero-order correlations, the relationships of the HQ factors with the hypothesized food-related convergent measures.

Finally, we compared the subdimension of HQ scores, expressed in mean values, by age, gender, genetic status, and weigh status groups. A series of Analyses of Variance (ANOVAs) were performed. First, we compared HQ scores by age-group. Then, a series of two-way ANOVAs with age group as one of the two fixed factors were performed to compare the HQ scores by gender, genetic status, and weight status. Interactions with age group were also investigated. Sidak's multiple comparisons *post-hoc* tests were performed after significant differences among means were determined.

## Results

### Inter-item Correlations

Overall, the examination of inter-item correlations highlighted significant high associations among items in the same hypothesized subscale (Supplementary Table 2). No inter-item correlations exceeded *r* ≥ 0.80, supporting the absence of content redundancy. However, one item in the Hyperphagic Behavior scale showed two marginally acceptable inter-items correlations (*r* = <0.30; “How often bargains, manipulates for more food”). Thus, it was removed from further analyses.

### Confirmatory Factor Analysis

The χ^2^ difference between the two hypothesized competitive model fits indicated that the three-factor model provides an adequate fit to the data compared to the one-factor model, $\Delta \chi_{\left(3\right)}^{2}$ = 56.92, *p* < 0.001. Although the χ^2^ was significant, $\chi_{\left(32\right)}^{2}$ = 79.79, *p* < 0.001, all the goodness-of-fit indices for the three-factor model reached acceptable values, χ^2^/df = 2.49; CFI = 0.96; SRMR = 0.04; RMSEA = 0.08 (90% CI: 0.06; 0.11). On the contrary, fit statistics for the one-factor model were definitely worse, $\chi_{\left(35\right)}^{2}$ = 136.71, *p* < 0.001; χ^2^/df = 3.91, CFI = 0.91; SRMR = 0.06; RMSEA = 0.12 (90% CI: 0.10; 0.14).

Based on the inspection of modification indices, the three-factor model could further improve by freeing the error covariances between item 7 and item 5, and between item 9 and item 11. Allowing the estimation of these parameters significantly improved the three-factor model, $\chi_{\left(30\right)}^{2}$ = 54.31, *p* < 0.004; χ^2^/df = 1.81, CFI = 0.98; SRMR = 0.04; RMSEA = 0.06 (90% CI: 0.03; 0.08), as demonstrated by the significant χ^2^ difference, $\Delta \chi_{\left(2\right)}^{2}$ = 25.48, *p* < 0.001.

**Figure 1:**
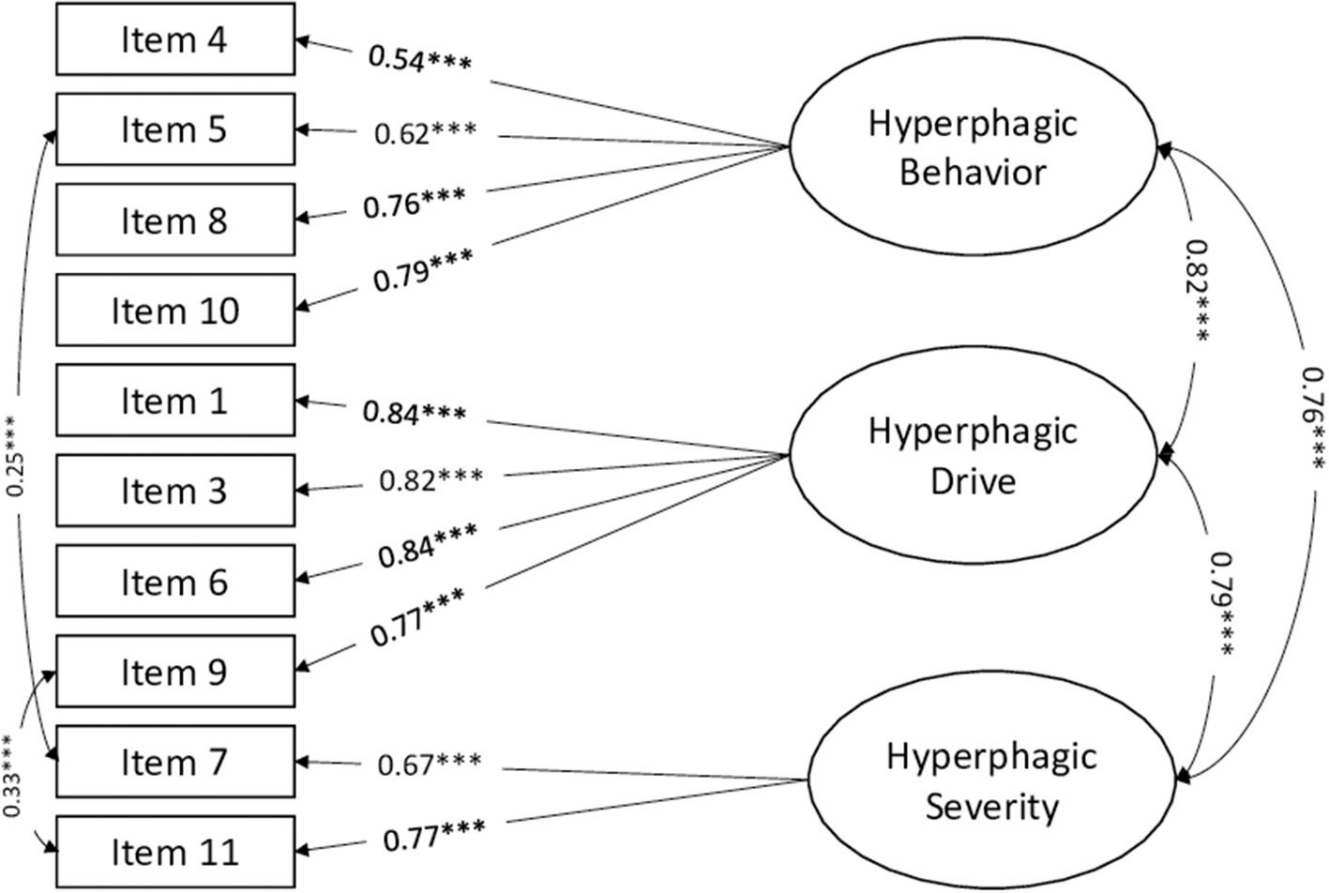
Standardized factor loadings and latent correlations among the three factors of the HQ. ***p < 0.001.

The standardized factor loadings and latent correlations for each of the three HQ factors are reported in Figure 1. Reliability coefficients demonstrated adequate internal consistency. More in detail, Cronbach's alphas were 0.73 for Hyperphagic Behavior, 0.89 for Hyperphagic Drive, and 0.66 for Hyperphagic Severity. McDonald's Omega coefficients were 0.81, 0.89, and 0.69 for Hyperphagic Behavior, Hyperphagic Drive, and Hyperphagic Severity, respectively.

### Factorial Invariance Across Age Groups

In order to investigate the hypothesis that the same three-factor structure is invariant across age groups (children vs. adults), we performed a series of multigroup structural equation modeling with hierarchical invariance tests. As can be observed from the comparison of fit statistics for invariance tests shown in Table 2, the fit of the three-factor model with the invariance of factor loadings across age groups (metric invariance) was not significantly different from that of the configural model. Further, the model estimating the invariance of intercepts across age groups (scalar invariance) showed a fit not statistically different from that of the metric model. Thus, it can be concluded that the factor structure of the HQ is invariant across age groups.

**Table 2:** Factorial invariance of the HQ across age groups.

	**χ^2^ (***df***)**	**Δχ^2^ (Δdf)**	***p*** **(Δχ^2^)**	**AIC**	**BIC**	**CFI**	**RMSEA (90% CI)**	**SRMR**
Configural model	88.83 (60)			5,681.99	5,918.91	0.97	0.06 (0.03–0.09)	0.05
Metric model (vs. configural model)	100.99 (67)	12.17 (7)	0.10	5,680.16	5,893.38	0.97	0.06 (0.04–0.09)	0.07
Scalar model (vs. metric model)	105.27 (74)	4.27 (7)	0.75	5,670.42	5,859.96	0.97	0.06 (0.03–0.08)	0.07

*Model comparisons. df , degrees of freedom; AIC, Akaike Information Criterion; BIC, Bayesian Information Criterion; Δχ^2^, difference in chi-square statistic between nested models; Δdf, difference in degrees of freedom between nested models; p(Δχ^2^), p-value of the Δχ^2^*.

### Association of HQ Factors With Food-Related Measures

HQ factors were positively associated, in both groups <18 ys>, to the hypothesized convergent measures represented by emotional overeating, food enjoyment, food responsiveness, and satiety responsiveness. Pearson's coefficients ranged from 0.37 to 0.73 (Table 3).

**Table 3:** Zero-order correlations between Hyperphagic dimensions and convergent variables distinguished by age group.

	**Behavior**		**Drive**		**Severity**	
	**<18 y**	**>18 y**	**<18 y**	**>18 y**	**<18 y**	**>18 y**
Emotional overeating	0.43	0.37	0.47	0.44	0.45	0.34
Food enjoyment	0.46	0.52	0.58	0.62	0.47	0.64
Food responsiveness	0.59	0.64	0.66	0.71	0.55	0.71
Satiety responsiveness	0.40	0.59	0.50	0.73	0.46	0.71

*All coefficients are significant (p < 0.001)*.

### Comparison by Age Groups, Sex, Genetic Status, Weight Status

**Figure 2:**
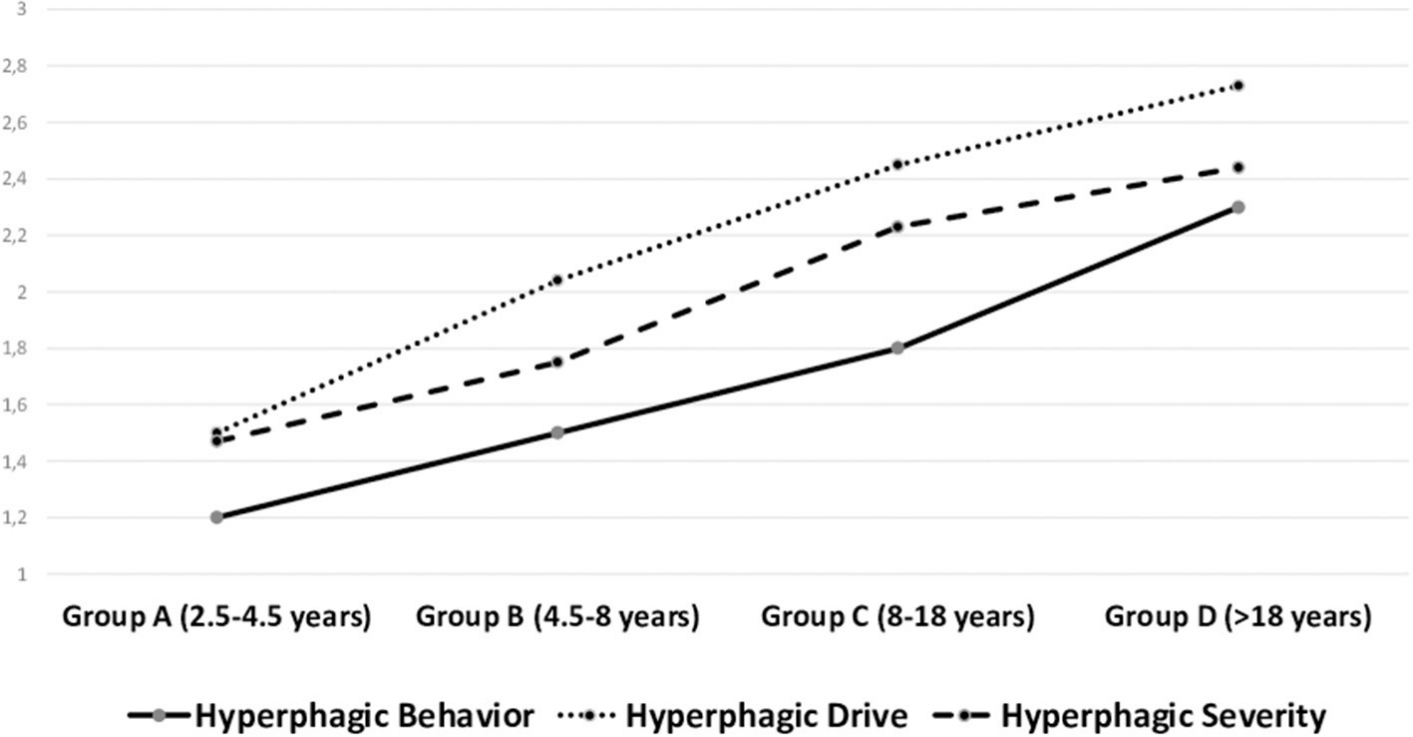
Comparisons of hyperphagia subdimensions by age-group. Values on the Y-Axis express the mean values of each dimension.

There was a significant main effect of age on Hyperphagic Drive [*F*_(3, 216)_ =10.96, *p* < 0.001; η^2^ = 0.13], Hyperphagic Behavior [*F*_(3, 216)_ = 16.52, *p* < 0.001; η^2^ = 0.19], and Hyperphagic Severity [*F*_(3, 216)_ = 6.51, *p* < 0.001; η^2^ = 0.08]. *Post-hoc* comparison revealed that Group A (2.1–4.5 years) showed lower scores in all three HQ scores than group C (8–18) and group D (>18); group B had lower scores in all three HQ scores than group D; group C had lower scores in Hyperphagic Behavior than group D. Differences by age groups are plotted in Figure 2.

Comparisons by sex, weight status and genetic status were performed maintaining age groups as control variable in order to take into account the variability due to the age-related nutritional phase.

No differences by sex emerged, neither interaction by age groups.

**Figure 3:**
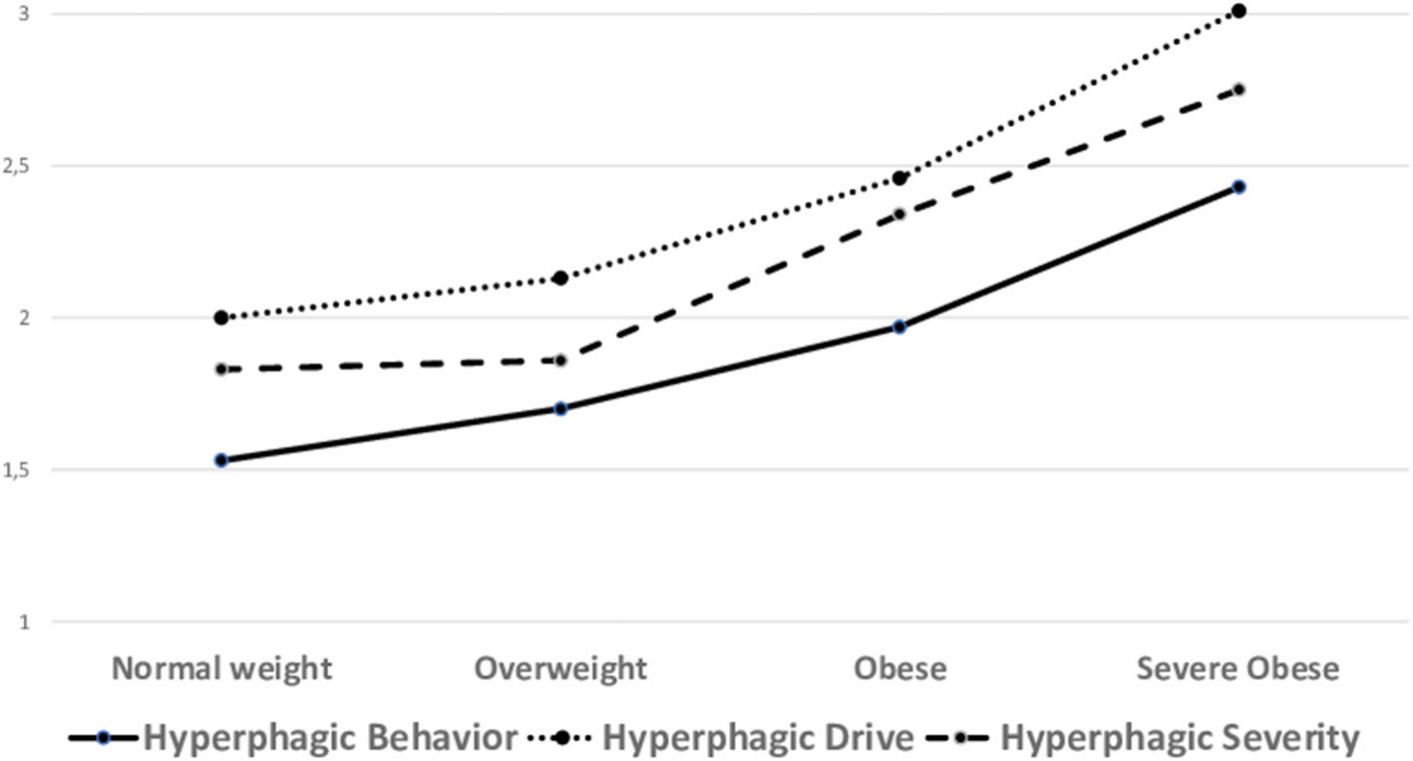
Comparisons of hyperphagia subdimensions by weight-status. Values on the Y-Axis express the mean values of each dimension.

There was a significant main effect of weight status on Hyperphagic Drive [*F*_(3, 216)_ = 7.10, *p* < 0.001; η^2^ = 0.09], Hyperphagic Behavior [*F*_(3, 216)_ = 5.94, *p* < 0.001; η^2^ = 0.08], and Hyperphagic Severity [*F*_(3, 216)_ = 4.31, *p* < 0.001; η^2^ = 0.06]. *Post-hoc* comparison revealed that in Hyperphagic Behavior and in Hyperphagic Drive, NW showed lower scores than OB and SOB group, OV were lower than SO, and OB were lower than SOB; in Hyperphagic Severity, NW and OV were lower than SOB. Non-interaction effect with age groups emerged. Differences by weight status are plotted in Figure 3.

**Figure 4:**
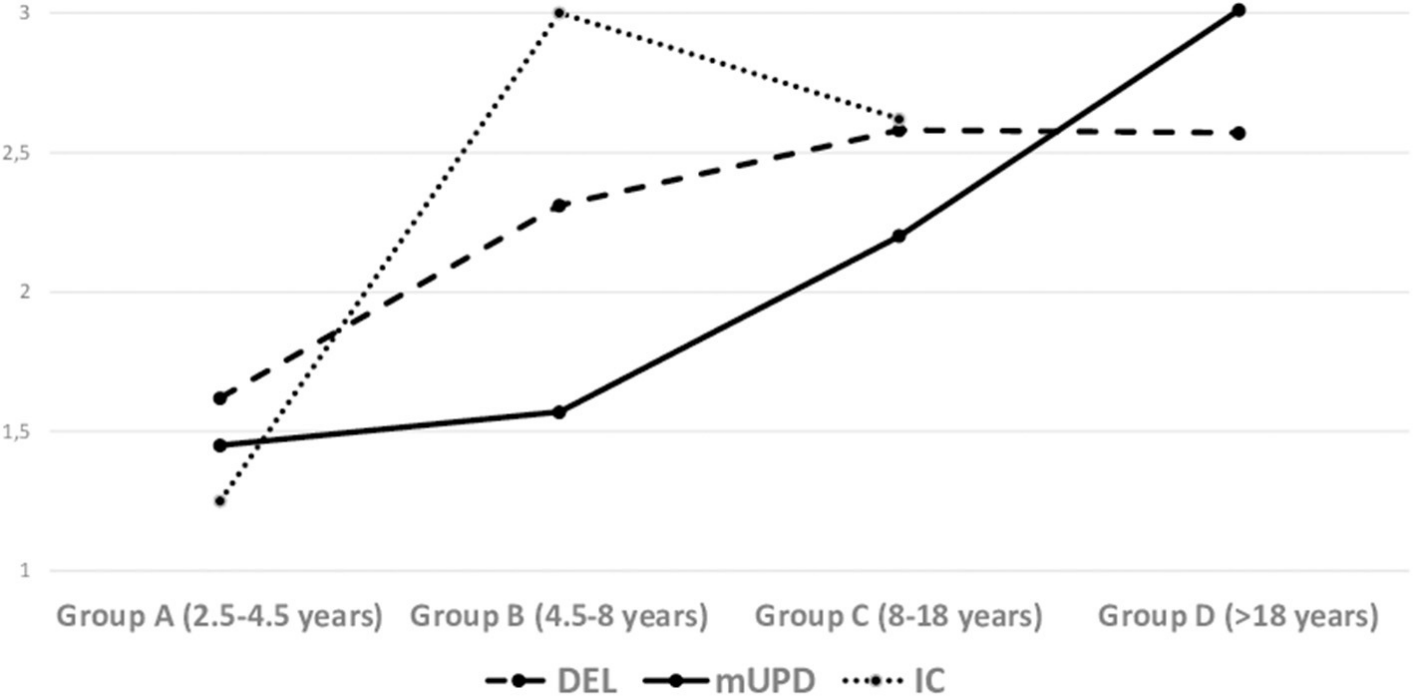
Interaction of age-group by genetic status in hyperphagic behavior. Values on the Y-Axis express the mean values of each dimension.

No differences emerged by genetic status whereas there was an interaction effect with age group for Hyperphagic Drive [*F*_(5, 211)_ = 2.98, *p* < 0.001; η^2^ = 0.07]. Means reported in Figure 4 showed that in DEL group, Dyperphagic Drive showed an early increase in the transition from phase A to phase B (age-related phases) and the stabilizes in the following phases, whereas in mUPD group Hyperphagic Drive showed a slower increase in the first years of life and an abrupt increase in the transition from phase B to phase C after age 8. Interaction effect is plotted in Figure 4. In Table 4 are reported means and standard deviations concerning each dimension by age, sex, genetic subtypes and weight status. Also a total score is reported computing the mean score after summing the 10-seleceted-items.

**Table 4:** Means and standard deviation of hyperphagic dimensions and total score by age groups, sex, genetic status, and weight status.

	**Drive**		**Behavior**		**Severity**		**Total score**	
	***M***	***SD***	***M***	***SD***	***M***	***SD***	***M***	***SD***
**Age groups**								
Group A	1.5	0.53	1.2	0.29	1.47	0.75	1.37	0.39
Group B	2.04	0.92	1.5	0.81	1.75	0.91	1.76	0.77
Group C	2.46	0.90	1.79	0.72	2.29	1.07	2.16	0.91
Group D	2.74	1.09	2.33	0.95	2.45	1.21	2.51	0.91
**Sex**								
Male	2.47	1.04	1.92	0.84	2.21	1.11	2.20	0.86
Female	2.36	1.01	1.89	0.94	2.18	1.10	2.13	0.87
**Genetic status**								
DEL	2.5	0.97	2.07	0.86	2.19	1.19	2.26	0.81
mUPD	2.26	1.05	1.75	0.88	2.11	1.17	2.02	0.90
IC	2.63	1.14	1.81	1.10	2.27	1.18	2.16	0.86
**Weight status**								
Normal weight	2.00	0.87	1.53	0.69	1.83	0.97	1.77	0.72
Overweight	2.14	0.91	1.71	0.76	1.86	1.07	1.90	0.76
Obese	2.46	0.91	1.97	0.87	2.31	1.14	2.23	0.78
Severe obese	3.07	1.11	2.43	0.95	2.75	1.05	2.75	0.86

*Values were computed dividing the total score of each dimension by the number of items composing the dimension*.

A detailed report of ANOVAs results is shown in Supplementary Table 3.

## Discussion

Hyperphagia is a hallmark of PWS. The HQ by Dykens et al. (15) is considered the effective tool to measure hyperphagia in individuals with PWS. Hyperphagic Behavior, Drive and Severity are the subdimensions extracted from the original 11 item- version of the HQ. Some studies confirmed the questionnaire structure whereas other suggested alternative solutions.

Our findings confirmed a three-factor solution corresponding to the three original subdimensions even if we dropped-out one item (“How often bargains, manipulates for more food”) because of weak correlation with the other items. The questionnaire evidenced a satisfactory invariance by age (<18>) and a convergent validity with measures concerned proximal food-related behaviors and attitudes.

Having a valid and reliable measure for assessing hyperphagia is of crucial importance in individuals with PWS both in the diagnostic process and in the treatment. Despite clinical correlates of PWS seem stably recurrent, hyperphagic symptoms highly varies among individuals and changes in hyperphagia are significant markers of the efficacy of the treatment.

Regarding to the second aim of the research, the comparison of the hyperphagic scores by age, gender, weight status and genetic subtypes partially confirmed our hypotheses. As we expected, all the three subdimension of the hyperphagia tend to linearly increase during the developing years. Lower levels characterize the first group of age from 2.5 to 4.5 years when hyperphagia is not fully manifest even though metabolic disorders begin to manifest. An increase of hyperphagia occurs in the second period from 4.5 to 8 years to reach a further increasing in the third period from 8 to 18 years. Nevertheless, we did not find a mitigation of hyperphagia in the adult group, since a further increase of the levels of hyperphagia occurred, especially of the Hyperphagic Behavior. Despite the great audience received in the literature about the assumption that hyperphagia decreases and becomes more treatable in the adult age (12), other recent studies did not find differences in hyperphagia among age groups (11, 38, 39). Similarly, also Dykens et al. (15) even differently grouping participants by age, did not find a decrease of hyperphagia in older age. We are aware that methodologically it is a hazard to compare individuals of so different age, without controlling life circumstances and the environmental interplay on genetic characteristics (40). An historical effect due to new therapeutic strategies and new cultural attitudes toward the disease might significantly affect the responses to the disease both in individuals with PWS and their caregivers. It should be also considered that the older age of the parents of adults with PWS could interfere with a more effective control on their child behavior. However, this issue might be further examined.

Our findings evidenced that hyperphagia is more pronounced in individuals with PWS with higher weight status even accounting for the age-group. This result may seem obvious, since weight status is a direct effect of hyperphagia, but it should be noted that within each age-group obesity is variably distributed, suggesting again that individual responses to the syndrome can be highly variable (38).

We did not find a main effect of the genetic status on hyperphagia., in agreement with a previous study (15). However, we found an interaction effect between genetic status and age-group in the Hyperphagic Drive. A different trend across age emerged for DEL vs. mUPD group (we avoided inference analyses in the IC group for the small number of cases). From 2.5 to 4.5 years both DEL and mUPD groups showed low scores of Hyperphagic Drive, while from 4.5 to 8 years DEL group showed a sharp increasing and became stable in the following years. Conversely, PWS individuals with mUPD showed a stability of Hyperphagic Drive from the first period to the second period and exhibited a sudden increasing after the 8 years until the adult age. The existence of two different tempos of Hyperphagic Drive in function of the genetic cause requires further investigation but might shed a new light on the genetic mechanisms of hyperphagia.

### Strengths and Limitations

This is the first systematic study carried out on hyperphagia in Italian individuals with PWS. The sample is highly representative (by gender and age) since the recruitment involved the main Clinical Units of PWS in the Italian country. The affiliation of the researchers to the same scientific society has guaranteed a high uniformity in terms of procedures. The study also presents a series of limitations, such as the lack of test-retest investigation to consider the stability of the responses across time. In addition, information related to the parenting practices in exerting a control on dysfunctional behavior of their children was missing. Lastly, the use of a cross-sectional research design does not allow to conclude whether differences among individuals in function of the age-groups are due to maturational processes, pharmacological treatments or psycho-educational approaches. A further critical issue of the HQ concerns the presence of different timeframes for caregiver recall of the hyperphagic symptoms across the HQ questions. To overcome this limitation, the 9-item version (HQ-CT) (21) sets the time interval to the last 2 weeks.

### Implications and Future Directions

The availability of a valid measure to assess hyperphagia represents an additional tool for RCT aimed to evaluate the effectiveness of intervention protocols. Reducing hyperphagia should be a goal in the treatment of individuals with PWS whose physical health is seriously compromised by an excessive weight gain. Moreover, their quality of life could significantly improve limiting the persistent and compulsory search of food (20). To date, no effective treatment for managing hyperphagia has been found even though several experimental protocols have been assessed (22). Future research might extend the assessment of hyperphagia (using the HQ) to other disorders characterized by dysfunctional eating behaviors, allowing a more accurate clinical assessment. Also, future research should be addressed to the role of genetic subtypes in the development of hyperphagia to improve our knowledge about the biological and psychological components of this dysfunctional behavior.

## Data Availability Statement

The original contributions presented in the study are included in the article/Supplementary Materials, further inquiries can be directed to the corresponding author/s.

## Ethics Statement

The studies involving human participants were reviewed and approved by Ethical Committee of the Santobono-Pausilipon Hospital (leader center of the study) and by the Review Board of the Genetic Obesity Study Group of the Italian Society of Pediatric Endocrinology and Diabetology. The patients/participants provided their written informed consent to participate in this study.

## Author Contributions

DB, ML, and GV designed the study. ML, AC, DF, GG, SO, LR, MS, LI, LD, AF, MW, MF, and MD conducted the study. DB and CE analyzed the data. DB and ML interpreted the data and drafted the manuscript. GV contributed to literature review, conceptualization of the findings, and provided critical revisions to the manuscript. All authors contributed to the article and approved the submitted version.

## Funding

This work was supported by SIEDP.

## Conflict of Interest

The authors declare that the research was conducted in the absence of any commercial or financial relationships that could be construed as a potential conflict of interest.

## Publisher's Note

All claims expressed in this article are solely those of the authors and do not necessarily represent those of their affiliated organizations, or those of the publisher, the editors and the reviewers. Any product that may be evaluated in this article, or claim that may be made by its manufacturer, is not guaranteed or endorsed by the publisher.
