# Supplementary material for: The Hyperphagia Questionnaire: Insights From a Multicentric Validation Study in Individuals With Prader Willi Syndrome
Source: Front Pediatr. 2022 Feb 14;10:829486. doi: 10.3389/fped.2022.829486 (PMC8884358; doi:10.3389/fped.2022.829486)
Supplement: Supplementary file 2 [file Table_1.docx]

Table 1S

Italian Transalation of the Hyperphagia Questionnaire ^15^

| (1) In generale, qual è il grado di contrarietà di suo figlio quando il cibo che desidera gli viene negato?  ___ Non particolarmente contrariato  ___ Un pò contrariato  ___ Abbastanza contrariato  ___ Molto contrariato  ___ Estremamente contrariato |
| --- |
| (2) Con che frequenza suo figlio cerca di contrattare o manipolare le persone per ottenere più cibo durante i pasti?  ___ Qualche volta l'anno  ___ Qualche volta al mese  ___ Qualche volta la settimana  ___ Più volte la settimana  ___ Più volte al giorno |
| (3) Una volta che suo figlio pensa al cibo, è facile per lei o per le altre persone distogliere la sua attenzione dal cibo e rivolgerla verso altre cose?  ___ Estremamente facile, richiede un minimo sforzo  ___ Molto facile, richiede solo un po’ di sforzo  ___ Abbastanza difficile, richiede un certo sforzo  ___ Molto difficile, richiede molto lavoro  ___ Estremamente difficile, richiede un lavoro costante e difficile |
| (4) Con quale frequenza suo figlio cerca cibo nella spazzatura?  ___ Mai  ___ Qualche volta l'anno  ___ Da una a due volte al mese  ___ Da una a tre volte la settimana  ___ Da quattro a sette volte la settimana |
| (5) Con quale frequenza suo figlio si alza di notte in cerca di cibo?  ___ Mai  ___ Qualche notte l'anno  ___ Da una a due notti al mese  ___ Da una a tre notti la settimana  ___ Da quattro a sette notti la settimana |
| (6) Fino a che punto suo figlio può dimostrarsi perseverante nella richiesta o ricerca di cibo dopo che gli si dice "No" o "Non ne puoi avere di più! "?  ___ Abbandona rapidamente e facilmente la ricerca di cibo  ___ Abbandona abbastanza rapidamente e facilmente la ricerca di cibo  ___ È piuttosto perseverante nella sua ricerca di cibo  ___ È molto perseverante nella sua ricerca di cibo  ___ È estremamente perseverante nella sua ricerca di cibo |
| (7) Al di fuori degli orari dei pasti, per quanto tempo suo figlio a parla di cibo o ha comportamenti legati al cibo?  ___ Meno di 15 minuti al giorno  ___ Da 15 a 30 minuti al giorno  ___ Da 30 minuti a un'ora al giorno  ___ Da 1 a 3 ore al giorno  ___ Più di 3 ore al giorno |
| (8) Con quale frequenza suo figlio cerca di rubare il cibo (che lei sappia)?  ___ Qualche volta l'anno  ___ Qualche volta al mese  ___ Qualche volta la settimana  ___ Più volte la settimana  ___ Più volte al giorno |
| (9) Quando gli altri cercano di impedire a suo figlio di parlare di cibo o di avere comportamenti legati al cibo, ciò generalmente causa:  ___ Nessun malessere o contrarietà  ___ Un leggero malessere o contrarietà  ___ Un malessere o contrarietà moderati  ___ Un malessere o contrarietà gravi  ___ Un malessere estremo; non è generalmente possibile fermare questi comportamenti. |
| (10) Fino a che punto suo figlio si può mostrare scaltro o svelto nell’ ottenere il cibo?  ___ Non particolarmente  ___ Leggermente  ___ Abbastanza  ___ Molto  ___ Moltissimo |
| (11) Fino a che punto i pensieri, i discorsi o i comportamenti legati al cibo interferiscono con la routine quotidiana le sue cure personali, la scuola o il lavoro di suo figlio?  ___ Nessuna interferenza  ___ Interferenza lieve: interferenza occasionale legata al cibo nel compimento dei compiti scolastici, del lavoro o dell'igiene personale  ___ Interferenza moderata: interferenza frequente legata al cibo nel compimento dei compiti scolastici, del lavoro o dell'igiene personale  ___ Interferenza grave: interferenza quasi quotidiana legata al cibo nel compimento dei compiti scolastici, del lavoro o dell'igiene personale  ___ Interferenza estrema: spesso non è in grado di partecipare ai compiti di igiene personale, o di recarsi a scuola o al lavoro a causa delle difficoltà legate al cibo |
