# Supplementary material for: The Hyperphagia Questionnaire: Insights From a Multicentric Validation Study in Individuals With Prader Willi Syndrome
Source: Front Pediatr. 2022 Feb 14;10:829486. doi: 10.3389/fped.2022.829486 (PMC8884358; doi:10.3389/fped.2022.829486)
Supplement: Supplementary file 3 [file Table_2.docx]

| Table 2S  *Inter-item correlations.* | | | | | | | | | | | |
| --- | --- | --- | --- | --- | --- | --- | --- | --- | --- | --- | --- |
|  | Q2 | Q4 | Q5 | Q8 | Q10 | Q1 | Q3 | Q6 | Q9 | Q7 | Q11 |
| *Hyperphagic Behavior* |  |  |  |  |  |  |  |  |  |  |  |
| 2. How often bargains, manipulates for more food | 1 |  |  |  |  |  |  |  |  |  |  |
| 4. How often forages through trash for food | 0.30*** | 1 |  |  |  |  |  |  |  |  |  |
| 5. How often gets up at night to seek food | 0.30*** | 0.51*** | 1 |  |  |  |  |  |  |  |  |
| 8. How often tries to steal food | 0.58*** | 0.46*** | 0.47*** | 1 |  |  |  |  |  |  |  |
| 10. How clever or fast in obtaining food | 0.51*** | 0.37*** | 0.44*** | 0.61*** | 1 |  |  |  |  |  |  |
| *Hyperphagic Drive* |  |  |  |  |  |  |  |  |  |  |  |
| 1. How upset when denied food | 0.61*** | 0.32*** | 0.39*** | 0.49*** | 0.56*** | 1 |  |  |  |  |  |
| 3. Once food on mind, how easy to redirect  away from food | 0.52*** | 0.30*** | 0.43*** | 0.46*** | 0.56*** | 0.71*** | 1 |  |  |  |  |
| 6. How persistent in asking or looking for food  when told no | 0.62*** | 0.39*** | 0.46*** | 0.56*** | 0.58*** | 0.70*** | 0.69*** | 1 |  |  |  |
| 9. Level of distress when others stop food talk  or behaviors | 0.49*** | 0.28*** | 0.43*** | 0.47*** | 0.56*** | 0.66*** | 0.63*** | 0.63*** | 1 |  |  |
| *Hyperphagic Severity* |  |  |  |  |  |  |  |  |  |  |  |
| 7. Time spent talking about food or engaged in  food behavior | 0.43*** | 0.22*** | 0.46*** | 0.42*** | 0.44*** | 0.42*** | 0.40*** | 0.46*** | 0.41*** | 1 |  |
| 11. Time spent talking about food or engaged in food behavior | 0.54*** | 0.18** | 0.36*** | 0.42*** | 0.52*** | 0.52*** | 0.52*** | 0.50*** | 0.61*** | 0.51*** | 1 |

*Notes*. ****p* < 0.001, ***p* < 0.01
