# Supplementary material for: The Hyperphagia Questionnaire: Insights From a Multicentric Validation Study in Individuals With Prader Willi Syndrome
Source: Front Pediatr. 2022 Feb 14;10:829486. doi: 10.3389/fped.2022.829486 (PMC8884358; doi:10.3389/fped.2022.829486)
Supplement: Supplementary file 4 [file Table_3.docx]

**Table 3S**

*Comparison by ANOVA of hyperphagia by age groups, gender, genetic status, weight status, parental education level, and interaction between gender, weight status, genetic status, parental education level with age groups.*

|  | Drive | | Behavior | | Severity | |
| --- | --- | --- | --- | --- | --- | --- |
|  | *F* | *ƞ*^2^ | *F* | *ƞ*^2^ | *F* | *ƞ*^2^ |
| Age groups | 10.96*** | 0.13 | 16.52*** | 0.19 | 6.51*** | 0.08 |
| Sex | 0.001 | 0.01 | 0.04 | 0.01 | 0.28 | 0.01 |
| Sex*age groups | 1.24 | 0.02 | 0.94 | 0.01 | 1.04 | 0.01 |
| Weight Status | 7.10*** | 0.09 | 5.94** | 0.08 | 4.31** | 0.06 |
| Weight Status* age groups | 2.98* | 0.07 | 1.01 | 0.02 | 1.04 | 0.02 |
| Genetic Status | 1.18 | 0.01 | 0.73 | 0.01 | 1.06 | 0.01 |
| Genetic status by age groups | 2.98* | 0.07 | 1.01 | 0.03 | 1.04 | 0.03 |

*Notes*. * *p* < 0.05, ** *p* < 0.01, *** *p* < 0.001
